# Dissemination and Implementation of a Google Apple Exposure Notification System for COVID-19 Risk Mitigation at a National Public University: Protocol for a Pilot Evaluation Study in a Real-World Setting

**DOI:** 10.2196/32567

**Published:** 2022-01-19

**Authors:** Cathy Lee Melvin, Katherine Regan Sterba, Ron Gimbel, Leslie Andrew Lenert, Kathleen B Cartmell, SC Safer Together Team

**Affiliations:** 1 Department of Public Health Sciences College of Medicine Medical University of South Carolina Charleston, SC United States; 2 College of Behavioral, Social and Health Sciences Clemson University Clemson, SC United States; 3 See Authors' Contributions United States

**Keywords:** COVID-19, risk, mitigation, mobile phone technology, exposure notification system, university setting, implementation science, implementation, dissemination, notification, university, exposure, transmission, communication, strategy, outcome, acceptability, adoption, usage

## Abstract

**Background:**

As SARS-CoV-2, the virus that causes COVID-19, spread rapidly across the United States in the spring of 2020, institutions of higher education faced numerous challenges associated with minimizing risk of exposure to COVID-19 among their students, faculty, staff, and surrounding communities. This paper describes the protocol, South Carolina (SC) Safer Together, developed by Clemson University (Clemson) to design, deploy, and evaluate multi-level communication and dissemination and implementation (D&I) strategies in line with recommendations from governmental and educational agencies to mitigate the risk of exposure to COVID-19. Safer Together was enhanced by the addition of the Google/Apple Exposure Notification app, an alternative strategy to support a recommendation of COVID-19 testing outcomes: contact tracing, isolation, and quarantine.

**Objective:**

This study aimed to (1) describe the content and intended audiences of D&I strategies used to deploy recommended COVID-19 mitigation strategies on a major college campus; (2) determine the reach, acceptability, adoption, and use of D&I strategies among target audiences among university students, faculty, and staff; and (3) characterize barriers and facilitators to the implementation and use of recommended mitigation strategies.

**Methods:**

The study team incorporated elements of the Health Belief Model, the Technology Acceptance Model, communication and social marketing models, and the Reach, Effectiveness, Adoption, Implementation, and Maintenance (RE-AIM) framework to identify and develop appropriate constructs and specific outcomes for inclusion in our approach to evaluate the communication, dissemination and implementation processes related to deployment of Safer Together at Clemson. A parallel convergent mixed methods design was used to (1) inform implementation strategies used to launch the program and (2) evaluate program reach, acceptability, adoption, and use guided by the RE-AIM framework. Data collection tools include surveys, data analytics–tracking, and focus groups or interviews with key stakeholders (students, employees, and university leadership).

**Results:**

Rigorously studying both the dissemination and implementation of Safer Together in a national public university setting is expected to yield lessons that will be valuable at many organizational and governmental settings. On a local level, broad adoption and use of the Safer Together may help reduce COVID-19 transmission and keep the university “open.” On a larger scale, lessons learned on how to influence student and employee behavior with respect to the use of a public health outbreak prevention tool including Safer Together may be applicable in future pandemic and outbreak situations.

**Conclusions:**

This study proposes a structured, theory-driven approach to evaluate dissemination and implementation strategies associated with the deployment of Safer Together in a university setting from the viewpoint of students, employees, and university leadership. Our results will inform future implementation of apps such as Safer Together at major state universities in SC.

**International Registered Report Identifier (IRRID):**

DERR1-10.2196/32567

## Introduction

### Background

As SARS-CoV-2, the virus that causes COVID-19, spread rapidly across the United States in the spring of 2020, institutions of higher education (IHEs) faced numerous challenges associated with minimizing the risk of exposure to COVID-19 among their students, faculty, staff, and surrounding communities [1-3].

This paper describes the protocol used by Clemson University (hereinafter referred to as “Clemson”) to design, deploy, and evaluate multi-level communication and dissemination and implementation (D&I) strategies in line with recommendations from governmental and educational agencies to mitigate the risk of exposure to COVID-19 and to assess D&I outcomes (reach, acceptability, adoption, and use), barriers, and facilitators encountered during this deployment from the perspective of multiple stakeholders.

Published guidelines of the Centers for Disease Control and Prevention (CDC) [4-7], the Equal Employment Opportunity Commission [8], the US Department of Education on the Family Educational Rights and Privacy Act [9], the Health Insurance Portability and Accountability Act [10], state and local health agencies and local governments, and various educational organizations such as the Chronicle of Higher Education [11] included these specific strategies for consideration by IHEs:

1. Opening in various formats (eg, virtual only or a hybrid of virtual and in-person classes) during the spring and fall semesters of 2020
2. Requiring entry screening prior to the beginning of each term
3. Implementing a universal screening testing strategy based on whether community SARS-CoV-2 was deemed moderate, substantial, or high
4. Assuring the availability of sufficient testing capacity
5. Consulting with local public health authorities
6. Implementing actions to support testing outcomes of contact tracing, isolation, and quarantine
7. Providing options to immediately separate students with COVID-19 and their close contacts by providing virtual learning options and self-isolation and self-quarantine rooms in residence halls or other housing facilities.
8. Providing support to students to manage COVID-19 symptoms, including medical care as necessary, as well as support managing emotional issues related to isolation or quarantine
9. Provision of alternative food service arrangements for students living on campus
10. Offering alternative teaching and work-at-home options for faculty, instructors, and staff who have COVID-19 or have been identified as a close contact, provided that they are well enough to continue working remotely, and
11. Considering the implementation of flexible sick leave and supportive policies and practices.

Clemson developed and implemented a plan that addressed each of these recommended strategies, in some form, in spring 2020.

Contact tracing is one typical and vital public health response to identify and isolate exposed close contacts (ECC) of known COVID-19 cases. The infectivity characteristics of SARS-CoV-2, the large proportion of very severe or deadly cases, and combined lack of specific treatment options for SARS-CoV-2 make it imperative to quickly isolate infected individuals and identify and track their ECC to reduce the odds of continued spread [12]. Moreover, for SARS-CoV-2, the incubation period is short (ie, 3-5 days); hence, chains of transmission must be recognized quickly in order to have an impact [12,13].

Implementing a timely contact tracing process is relatively slow, requires multiple calls to track down described contacts for interviews, and is labor-intensive. Simulation studies suggest the process is too slow to meaningfully limit COVID-19 spread [14]. In actual practice, reaching out to identified contacts is often unsuccessful [13,14]. Furthermore, the resulting data are flawed by memory biases, and many “contacts” never know that they were exposed.

To circumvent these issues, conventional contact tracing includes asking “cases” about where they were (ie, settings of potential exposure). While this information can be helpful in identifying ECC, information regarding the distance between persons and the total time of exposure gathered in this process often results in some individuals being considered as ECC when they actually had very limited or no exposure to an infected individual.

The time delays and personal recall limitations inherent in conventional contact tracing as well as the epidemiology and pathogenesis of SARS-CoV-2 provide a rationale for investigating novel approaches to control infection via contact tracing such as mobile apps [15].

To address the recommended strategy of implementing actions to support testing outcomes such as contact tracing, isolation, and quarantine, Clemson also took advantage of a novel partnership among the state public health authority for South Carolina (SC), the SC Department of Health and Environmental Control (DHEC), Google, and Apple.

**Figure 1:**
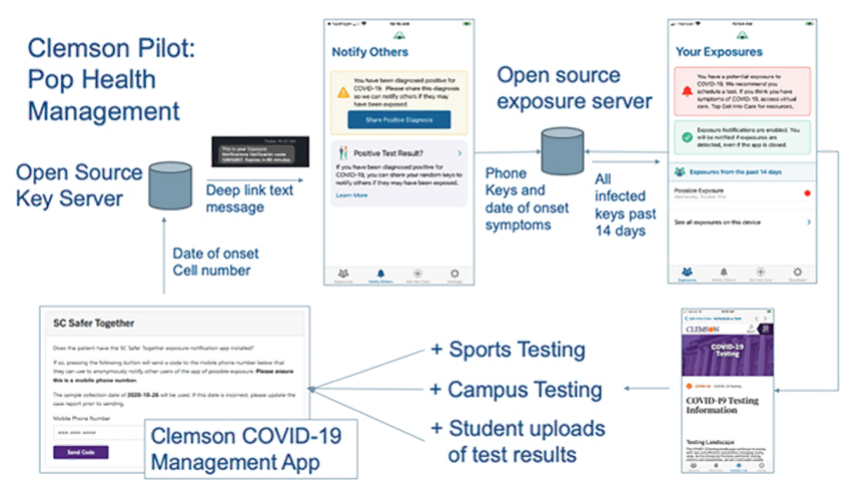
Clemson University's information ecosystem for Safer Together.

In 2020, Google and Apple created the Google/Apple Exposure Notification system (GAEN), a novel mobile app to help governments and the global community accelerate the process of contact tracing [16]. Mutual agreements among the DHEC, Google, Apple, Clemson Computing and Information Technology (CCIT), and the Medical University of South Carolina (MUSC) Biomedical Informatics Center branded GAEN as the SC Safer Together App (hereinafter referred to as “Safer Together”). Safer Together became part of an existing informational ecosystem at Clemson, which links test-ordering, test result–reporting, and case referral and management with exposure notification. Combined with deployment of other recommended strategies for COVID-19 mitigation, Safer Together was adopted as an efficient and privacy-protected approach to contact tracing (Figure 1). This system of notification, sharing of test results, and rapid access to testing or medical care when notified of exposure was expected to minimize contact tracing time and conserve campus resources.

Safer Together interfaces with public health, clinical providers, and clinical laboratory systems as the source of official laboratory findings. Case managers at Clemson Redfern Health Center use an automated function to send the text message, which allows the Key Authorization Server to deliver a digital certificate to the user’s cellphone. The cellphone user chooses whether they upload their test result to the exposure server. 

To assure user confidentiality, each phone advertises itself using multiple keys that rotate over time to protect from malfeasants. If a match is found with another phone, the duration of exposure (total of 15 minutes at less than 6 feet over 24 hours) and the signal strength are evaluated by functions built into Safer Together and aligned with revised CDC guidelines for significant exposures [6,16].

When an exposure notification is triggered, the exposed individual can use the “get into care” tab for information on what to do next. Compared to conventional contact tracing based on “case” recollection, cellphone-based exposure notifications have the potential to inform individuals of exposures more quickly, to identify a more complete set of true contacts, and to better assess extent of exposure [12]. Additionally, privacy-preserving Bluetooth technology promptly provides contacts with information regarding the frequency and intensity of lower-grade (shorter time, further distant) exposures, thereby empowering users to proactively measure their risks and take actions, which might safeguard their own health and public health, such as obtaining a test to determine their COVID-19 status or voluntarily quarantining.

Clemson operational units had concerns about whether their campus had sufficient beds and capacity to manage anticipated increases in quarantine facility requirements, based on Safer Together identification of ECC. A compromise plan was developed to conduct a limited pilot deployment of Safer Together for approximately 380 on-campus students living in 2 dormitories.

The first round of student messaging regarding recommended strategies and the use of Safer Together was initiated through Clemson Student Housing on October 28, 2020, to students in the two dormitories. On January 9, 2021, Safer Together was messaged and launched with all Clemson students in 2 waves, the first with students on campus and the second with all students regardless of residence.

**Figure 2:**
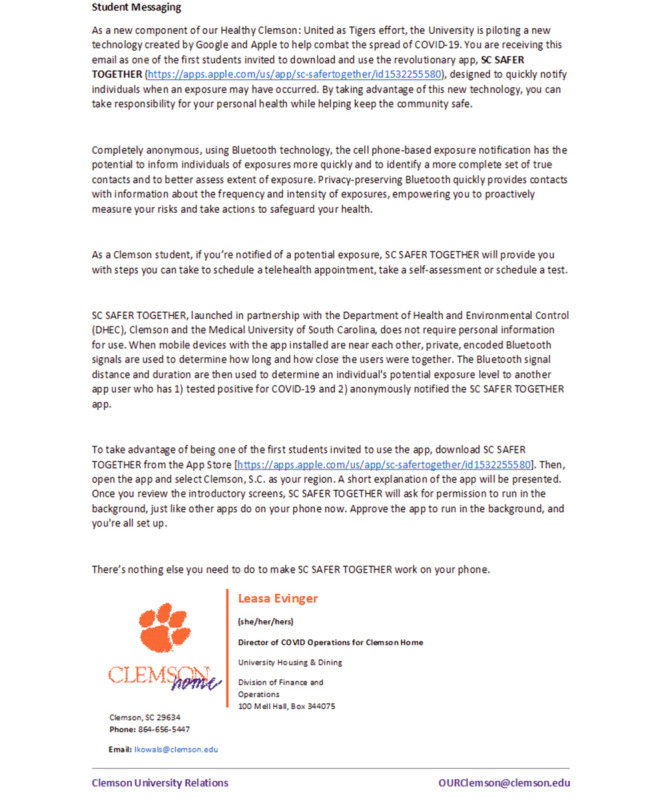
Student messaging.

Messaging for students was sent via email to explain Safer Together and its capacity to let students know if they had been exposed to someone with confirmed COVID-19, encourage them to download the app, link the app to the Healthy Clemson United as Tigers app, and understand what to do should she/he be notified of potential exposure. Students were also encouraged to participate in efforts to evaluate deployment strategies and learn about their acceptance and use of Safer Together (Figure 2).

**Figure 3:**
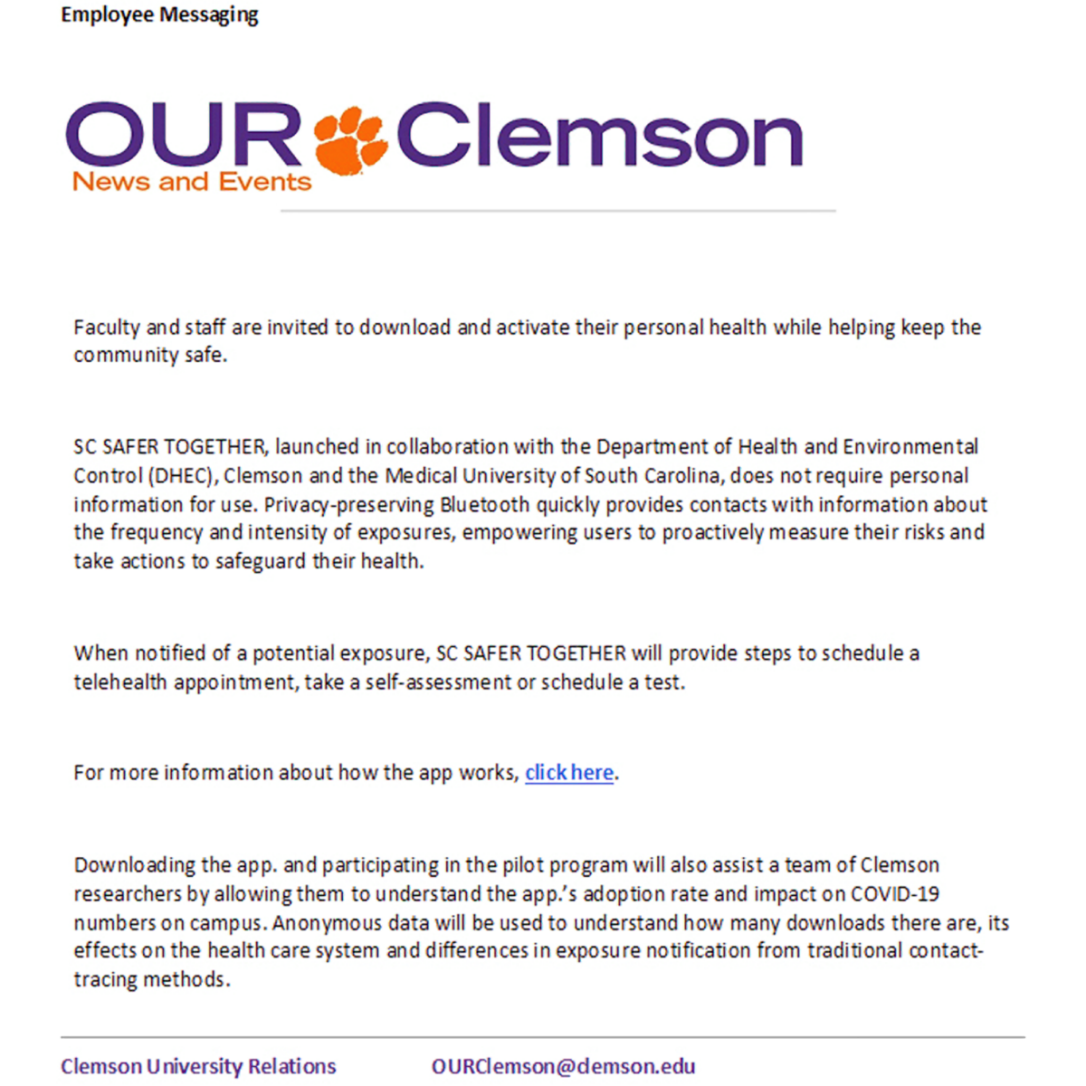
Employee messaging.

Given the small size of this pilot with students in only 2 dormitories and that on-campus isolation was not a burden to the university employees, Clemson’s administration disseminated messaging about Safer Together to all Clemson employees on November 20, 2020, via an email asking them to download and activate Safer Together and advising them of the effort to evaluate Safer Together deployment strategies (Figure 3).

### Evaluation Process Infrastructure

The D&I team is composed of individual representatives of Clemson Public Health Sciences, Clemson Human Resources, Clemson Student Affairs, Clemson Housing, Clemson Strategic Communications, MUSC College of Medicine Department of Public Health Sciences, Clemson Redfern Health Center, and Clemson Strategic Communications. This team was responsible for developing the study protocol, gaining institutional review board (IRB) approvals at both Clemson and MUSC, developing surveys and focus group and interview guides, and coordinating all data collection with Clemson Human Resources, Clemson Redfern Health Center, and Clemson Strategic Communications.

The Clemson-MUSC Safer Together Implementation team was divided into three groups: D&I Research Group (11 members), Technology Design and Enablement (14 members), and Data Analysis (2 members).

The D&I Research Group includes 4 faculty members with expertise in D&I science and is responsible for all aspects of the evaluation study design.

The Technology Design and Enablement Group is composed of members of the MUSC Biomedical Informatics Center and Clemson CCIT. This team was responsible for developing Safer Together and coordinating all design components with systems at DHEC, Clemson Redfern Health Services, and relevant university service components.

The Data Analysis Group includes individuals from the Department of Clemson Public Health Sciences and the Department of Public Health Sciences at MUSC. This group was charged with creating and maintaining study-related databases and conducting analyses.

All teams met together weekly and, as needed, by team or group.

## Methods

### Specific Aims and Objectives

The purpose of this mixed methods study is to (1) to characterize and evaluate communication and D&I strategies used to promote and support the use of Safer Together and (2) to examine implementation outcomes (reach, acceptability, adoption, and use), barriers, and facilitators encountered from the perspective of multiple stakeholders.

The study objectives are to:

1. Describe the content and intended audiences of multi-level dissemination and implementation strategies used to deploy Safer Together and other recommended mitigation strategies
2. Determine the reach, acceptability, adoption, and use of Safer Together among targeted audiences of university students, faculty, and staff, and
3. Characterize barriers and facilitators to implementation and use of Safer Together and recommended other mitigation strategies.

### Evaluation Framework

Our D&I Research Group incorporated elements of the Health Belief Model (HBM) [17], the Technology Acceptance Model (TAM) [18], communication and social marketing models [19], and the Reach, Effectiveness, Adoption, Implementation, and Maintenance (RE-AIM) framework [20] to identify and develop appropriate constructs and specific outcomes for inclusion in our approach to evaluating the communication and D&I processes related to the deployment of Safer Together at Clemson.

The HBM was used create questions for use in surveys and focus groups about HBM constructs such as perceived susceptibility, perceived severity, perceived benefits, perceived barriers, and self-efficacy [17]. 

The TAM measures intent to use a new technology by assessing perceived usefulness, perceived ease of use, compatibility, and self-efficacy of mobile health care systems [18].

Communication and social marketing models were used to create messaging and expected outcomes and to influence behavior by offering target market members (in this case students and employees) an attractive package of benefits and by reducing barriers that would otherwise encourage (or discourage) them from engaging in the behavior [19].

The RE-AIM model served as the guiding framework for measuring the D&I of Safer Together in our research setting [20]. The RE-AIM framework allows multiple measures at various setting levels while focusing on the measurement of “real world” D&I outcomes and processes. Table 1 provides a conceptual overview of the RE-AIM framework as applied to the evaluation of Safer Together. 

**Table 1:** Conceptual overview of the Reach, Effectiveness, Adoption, Implementation, and Maintenance framework applied in Safer Together.

Framework concept	Reach	Adoption	Implementation
Outcomes	Number, proportion, and representativeness of students and employees participating in Safer Together	Number, proportion, and representativeness of settings and agents willing to initiate the Safer Together system	Extent to which the Safer Together system is launched, implemented, and used as planned
Data collection methods	Surveys and focus groups	Focus groups	Surveys, focus groups, and Data analytics tracking

### Study Design

A parallel convergent mixed methods design will be used to (1) inform implementation strategies (ie, marketing, distribution, and education) used to launch the program and (2) evaluate program reach, acceptability, adoption, and use guided by the RE-AIM framework [21]. The three main data collection tools include surveys, data analytics–tracking, and focus groups or interviews with key stakeholders (students, employees, and university leadership). Table 2 contains a more comprehensive description of the RE-AIM framework elements, key questions to be addressed, and data to be collected.

**Table 2:** Evaluation matrix based on the domains of the Reach, Effectiveness, Adoption, Implementation, and Maintenance framework.

Framework domain			Students or employees		Health care providers		University leaders
**Reach**							
	What proportion of eligible individuals were offered and then downloaded the Safer Together intervention?	✓		✓			
	What percent of individuals seeking a COVID-19 test report actively using Safer Together?	✓		✓			
	How did Safer Together use vary by individuals’ demographic characteristics (eg, age, race, gender, student vs employee, faculty vs staff, and student residence location)?	✓		✓			
**Effectiveness**							
	What number and percent of individuals seeking a COVID-19 test report receiving a risk exposure notification on Safer Together?	✓					
	Were there differences in the time of the risk exposure notification between Safer Together and formal COVID-19 contact tracing methods?	✓		✓			
	What was the effect of Safer Together on health care system outcomes (eg, work processes, organizational change, and interdisciplinary collaboration)?			✓		✓	
**Adoption**							
	What are the characteristics of setting and adopting Safer Together?			✓		✓	
	How well did Safer together fit with the values and expectations of stakeholders?	✓		✓		✓	
	How did the Safer Together system help Clemson University achieve their educational and practice missions during the pandemic?	✓		✓		✓	
**Implementation**							
	Was there sufficient leadership support and buy-in for the Safer Together system (predisposing)?	✓		✓		✓	
	What were the potential barriers to successful Safer Together implementation and use and how were they addressed (enabling)?	✓		✓		✓	
	What workflow adjustments were needed to streamline Safer Together into routines of daily life at Clemson University (enabling)?			✓		✓	
	What measures were needed to create readiness for Safer Together adoption, commitment, and buy-in by stakeholders (enabling)?			✓		✓	
	Did users perceive Safer Together as easy-to-use and useful (predisposing)?	✓		✓		✓	
	What were the confidentiality and data security concerns when adopting the Google/Apple Exposure Notification app and how were they addressed (enabling)?			✓		✓	
**Maintenance**							
	How did Safer Together use by stakeholders evolve over time?	✓		✓		✓	
	What efforts were needed to maintain the app participation rate and effectiveness?			✓		✓	
	Was the use of Safer Together sustained over time?			✓		✓	

### Study Setting

Clemson University, located in Upstate SC, is one of two land-grant universities in SC. Clemson was founded in 1889 and currently has 20,868 undergraduate students and 5538 graduate students. Clemson offers over 80 major and 90 minor areas of study and more than 130 graduate programs and maintains a 16:1 student to faculty ratio. As a major research university, Clemson was awarded US $106.3 million in research support in 2019-2020. A robust workforce of staff employees (n=4611), faculty employees (n=1742), and emeritus faculty (n=654), totaling to 6007 employees, supports all major initiatives and activities undertaken on and off campus.

### Participants

A convenience sample of Clemson students, employees, and leaders involved in implementing or facilitating the use of Safer Together will be presented the opportunity to participate in this study. The inclusion and exclusion criteria for this study are shown in Textbox 1.

**Study inclusion and exclusion criteria.**

**Inclusion criterion**

- All registered students and all employees (faculty and staff) of Clemson University

**Exclusion criteria**

- Being a nonemployee (eg, contractor)
- Not meeting the aforementioned inclusion criterion

### Participant Recruitment, Enrollment, and Consent

Recruitment strategies for each group using social marketing and targeted messaging are detailed below. University representatives from the Student Affairs, Human Resources, and Strategic Communications units will participate as consultants on the D&I team to ensure coordination of efforts.

Social marketing and targeted messaging strategies will be deployed throughout the duration of the study, with specific emphasis on three time points: initial return of students to campus, start of the spring semester (and flu season), and late spring before completion of the academic year.

Given the “opt-in” nature of these user choices, and the fact that identifiable data are not available to the research team, the research team received a waiver of informed consent.

Members of the D&I research team and the Administration department at Clemson will not know whether any user activates or allows deidentified sharing of COVID-19 test results.

With respect to student and employee survey participation, anonymous surveys will be undertaken to obtain information related to student and employee use and perceived usefulness of Safer Together. Accordingly, the D&I Team received a waiver of informed consent for the anonymous surveys to be administered through the academic year.

With respect to focus group discussions addressed below, the D&I Team received a waiver of written informed consent. Verbal consent will be obtained from all participants after a study investigator provides a description of the purpose, procedures, and risks and benefits of the study.

Potential participants include university students, employees, and leaders. Potential participants will be approached through targeted communication avenues (eg, student housing listservs and student and employee newsletters). Our D&I Team will verify that participants meet the study eligibility criteria and approach them via electronic media or cellphone-based methods. Participants will be considered to have been enrolled when verbal consent is obtained in focus groups and acknowledged by moving forward to complete the electronic survey.

### Implementation Strategies

Implementation strategies are designed to increase the uptake and use of the Safer Together App by students and employees.

### Social Marketing and Targeted Messaging

Social marketing seeks to influence behavior by offering target market members (in this case students and employees) an attractive package of benefits and by reducing barriers that would otherwise encourage (or discourage) them from engaging in the behavior.

Key elements of social marketing include mutual fulfillment of self-interests, consumer orientation, segmentation (marketing to various subsets of the organization), and a marketing mix (product, price, place, and promotion) [19]. The study D&I team (including consulting representatives of Clemson’s Strategic Communications, Student Affairs, and Human Resources units) will create targeted messaging based on social marketing principles to be delivered throughout the academic year 2020-2021.

The three major study time periods were the following: original back-to-school messaging in September-October 2020 timeframe, at the transition of semesters (and flu season) in the January-February 2021 timeframe, and spring break to the end of the academic year between March and May 2021.

The overall messaging campaign focused on (1) building a Clemson community partnership around COVID-19 infection control, (2) raising awareness about how exposure notifications can facilitate and supplement other public health efforts, (3) addressing privacy protections and security concerns, and (4) highlighting ease of use and voluntary participation.

Four main strategies were used for the campaign: (1) flyers were posted strategically across campus at the start of the semester; (2) presentations were made at key student, employee, and university leadership meetings; (3) email and social media messaging were used to provide strategic messages over the course of the planning, early implementation, and maintenance phases, and (4) university-level messaging was delivered electronically via selected venues.

Branding for all materials and messages allows consumers to identify and connect with messages. The Basic Communication Model [19] elements were used to guide the selection of optimal communication channels, sources, and messages to fit the target audiences of students and employees. Messages were distributed through highly visible channels and delivered by reputable sources (eg, Clemson leaders, staff, and student champions).  Table 3 provides further details about each of these strategies.

Messaging was designed to influence student and employee behavior and prompt use of Safer Together. Specific behaviors include downloading the app, activating the app, sharing test results on the app, and pursuing COVID-19 testing if a user receives a Safer Together exposure notification.

**Table 3:** Sample activities, content, and purpose for marketing activities.

Planned schedule of marketing activities	Content	Purpose
Flyers posted strategically on campus	Highlight the Clemson University community partnership around COVID-19 infection control; brief overview of the app and how to use it; topics will cover benefits of contact tracing, how exposure notification can help, privacy protection, and how the app works	To increase awareness and acceptance of the app, address potential user concerns, and inform users how to use it
Presentations at key web-based employee and student meetings	Highlight the Clemson University community partnership around COVID-19 infection control; brief overview of the app and how to use it; topics will cover benefits of contact tracing, how exposure notification can help, privacy protection, and how the app works	To increase awareness and acceptance of the app, address potential user concerns, and inform users how to use it
Email/social media blasts	Highlight awareness at multiple levels of the university, colleges, and departments; garner student support and enthusiasm	To increase awareness and commitment at the university and department levels
University messaging 1 (via selected venues)	Highlight the importance of university commitment to curtail the spread of COVID-19; expand the readership to a variety of stakeholders	Increase awareness to a broad range of stakeholders; convey commitment from university leadership
University messaging 2	Brief overview of the app and how to use it; topics will cover the importance of the Clemson university community partnership for COVID-19 infection control; the benefits of contact tracing, how exposure notification can help, privacy protection, and how the app works	To increase awareness and acceptance of the app, address potential user concerns, and inform users how to use it
University messaging 3	Reminder about Clemson University community partnership for COVID-19 infection control and frequently asked questions about the app, and who to contact with questions about installing and using the app if an exposure occurs	To address problems users may be having in using the app via a series of frequently asked questions
University messaging 4	Brief stories of how the app has been successful when used in other places	To increase awareness of public health benefits of using the app
University messaging 5	Brief update on Clemson University’s experience using the app and how it may help control the pandemic on campus	To increase buy-in regarding the app
University messaging 6	Reminder about Clemson University community partnership for COVID-19 infection control and engagement with the app again, repeating the brief overview	To provide a booster educational session to encourage continued use
University messaging 7	Messaging about not letting one’s guard down during the spring break—continue protections and engagement with the app (spring break starts on March 15)	To increase buy-in regarding the app during vacation
University messaging 8	Message of thanks for participating in the app, with a brief summary of Clemson University’s experience using the app	To debrief and increase buy-in

### Study Flow and Data Collection Processes

Closer to the date of student return to the Clemson campus, the university initiated a series of messages to students and employees about Safer Together and this study. Messaging encouraged downloading of Safer Together to student and employee cellphones, individual consent to activate Safer Together, and individual consent to share COVID-19 test results.

Students and employees selected through random sampling for COVID-19 testing, who present at the COVID-19 testing site at Clemson Redfern Health Center, will be asked questions as part of their registration process related to Safer Together. One question asks if the individual has downloaded and activated Safer Together. A second question relates to whether the individual presented for COVID-19 testing primarily because of a Safer Together exposure notification alert.

Students and employees not selected through random sampling but presenting for COVID-19 testing at the COVID-19 testing site will be asked questions as part of their registration process related to Safer Together. One question asks if the individual has downloaded and activated Safer Together. A second question relates to whether the individual presented for COVID-19 testing primarily because of a Safer Together exposure notification alert.

Students and employees were asked to complete a one-time electronic anonymous survey administered by the D&I team to address framework measures. Key questions included assessments of the individual’s decision to download and activate Safer Together, consent to record COVID-19 test results in Safer Together, and report whether they took some action in response to an exposure notification (eg, additional COVID-19 testing and quarantine). Additional questions assessed user experiences with the app and whether they found the app acceptable and easy to use. Among those who did not download the app, questions focused on factors influencing one’s decision to not download Safer Together, including perceived barriers to downloading the app. All participants were asked questions about testing behaviors, perceived susceptibility to and severity of COVID-19, and individual sociodemographic characteristics.

At key points during the study, focus groups facilitated by the D&I team will be conducted with key stakeholders including students and employees (health care providers and university leaders) to address framework measures. Questions will predominantly address measures within the Adoption, Implementation, and Maintenance domains of the RE-AIM framework by using a structured interview guide. Focus groups will be completed virtually (on Zoom), last approximately 45-60 minutes, and be audiotaped for analysis.

Key leaders involved in developing, planning, and launching the Safer Together initiative were selected to complete individual interviews using a structured interview guide. Participants will include representatives from the information technology office, the Clemson Redfern Health Center, the communications office, the Healthy Clemson: United as Tigers initiative, dormitory housing, and the public health strategy team. Participants will be invited to participate in a 30-minute, audiotaped interview.

**Figure 4:**
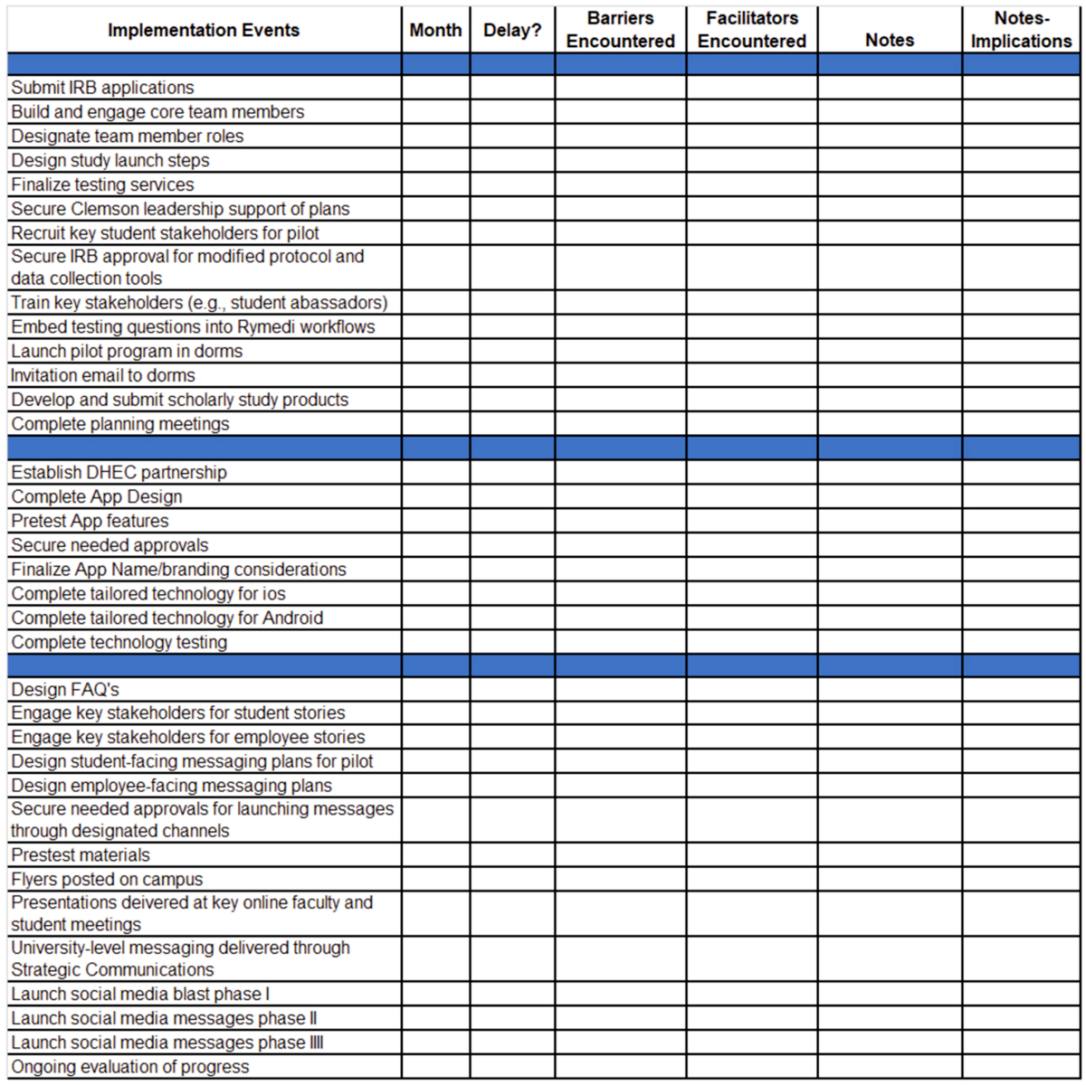
Implementation tracking log. DHEC: Department of Health and Environmental Control; FAQ: frequently asked questions; IRB: institutional review board.

Using a team-developed implementation tracking log (Figure 4), the D&I Team monitored the completion, timing, and barriers faced in accomplishing planning, engagement, technology development, and messaging milestones. The log was competed at routine team meetings to assess completion of steps (with “yes” or “no” responses) and whether delays (eg, information technology, communication, university leadership, and engagement) were encountered.

### Primary Outcomes of Effectiveness

In this study, limited data are available for extraction by the research team from Safer Together, so most study measures are captured by alternative approaches. The four primary outcome measures are downloading the Safer Together app, activating Safer Together, activating sharing of COVID-19 test results in Safer Together, and responsive behavior to Safer Together exposure notification.

#### Participants Downloading Safer Together

The number and proportion of research participants who seek testing and those who complete the survey and download Safer Together will be delivered via the DHEC to Clemson CCIT. We will record this statistic on a daily basis and aggregate for periods prior to and after our social marketing targeted messaging.

#### Participants Activating Safer Together

The number and proportion of research participants who present for COVID-19 testing and claim that they have downloaded Safer Together and activated its exposure notification alert will be recorded.

Participants who consent to sharing COVID-19 results within Safer Together and the number and proportion of those who declare that they have authorized the sharing of COVID-19 results on Safer Together will be recorded. These data are not available from Safer Together and can only be assessed through surveys and focus groups.

#### Effect of Safer Together Exposure Notification Alert on Participant Behavior

The number and proportion of research participants who present for COVID-19 testing and claim that the primary reason for presenting for COVID-19 testing was a Safer Together exposure notification alert will be recorded.

### Statistical Analysis

#### Sample Size

The survey was administered to a random stratified sample of 20% of Clemson employees (n=802) and students (n=4998), for a total sample size of 5790 individuals.

#### Analysis Plan

The analysis of our mixed methods approach will follow approaches for assessing and integrating findings from both a quantitative and qualitative perspective [21].

Safer Together overall use will be measured by a binary variable (“yes” or “no”). Univariate analyses for differences in app use via student demographics will be compared using chi-square tests and independent 2-sample *t* tests for categorical and continuous variables, respectively. Multivariate analyses will be performed using logistic regressions. Exposure notification time will be defined as the average of the time between an individual’s recorded COVID-19 test (if positive) and notification of all contacts. Differences in exposure time between the two methods will be assessed using Kaplan-Meier estimators and Cox proportional hazard models.

The reach of each method will be measured as the number of contacts informed; differences will be assessed using independent 2-sample *t* tests. Upon presentation to health services, students will be asked whether they were notified of exposure through the Safer Together app, formal contact tracing, or other. Comparisons between Safer Together app tracing and formal contact tracing will be conducted using chi-square tests.

Transcriptions of digital recordings of focus groups and field notes will be analyzed to characterize app use experiences and barriers and facilitators to program delivery, adoption, and implementation. Analysis will be conducted using manual coding to identify, categorize, and contextualize patterns. We will use an initial codebook derived from the RE-AIM framework also allowing additional themes to arise directly from the data. Two independent coders will read and reread transcripts, outlining and organizing themes and subthemes; discrepancies will be resolved in team meetings. After completing qualitative and quantitative data analyses independently, we will use graphical matrix configurations (“joint displays”) to integrate survey findings with qualitative data for data triangulation [21]. Qualitative themes will be supplemented by patterns identified in quantitative results guided by the RE-AIM framework.

We will use a team-based approach with individual and dual review of data and data findings with, as appropriate or needed, adjudication by a third party to interpret and translate results and determine D&I implications of our findings.

#### Ethics Approval

The protocol was approved by both the Clemson University IRB and the IRB-II-Medical University of South Carolina on September 11, 2020.

## Results

Rigorous evaluation of both the dissemination and implementation of Safer Together in a national public university setting is expected to yield insights that will be valuable at many organizational and governmental settings. On a local level, broad adoption and use of the Safer Together may help reduce transmission of COVID-19 and keep the university “open.” On a larger scale, lessons learned on how to influence student and employee behavior regarding the use of a public health outbreak prevention tool like Safer Together may be applicable in future pandemic and outbreak situations.

## Discussion

This study proposes a structured approach based on the RE-AIM framework, to evaluate dissemination and implementation strategies associated with deployment of the Safer Together in a university setting from the viewpoint of students, employees and university leadership. The results of this study will inform future implementation of apps such as Safer Together at major state universities.

## Abbreviations

CCIT: Clemson Computing and Information Technology
CDC: Centers for Disease Control and Prevention
DHEC: Department of Health and Environmental Control
D&I: dissemination and implementation
ECC: exposed close contacts
GAEN: Google/Apple Exposure Notification
HBM: Health Belief Model
IHE: institution of higher education
IRB: institutional review board
MUSC: Medical University of South Carolina
RE-AIM: Reach, Effectiveness, Adoption, Implementation, and Maintenance
SC: South Carolina
TAM: Technology Assessment Model
